# Dipeptidyl peptidase-4 is highly expressed in bronchial epithelial cells of untreated asthma and it increases cell proliferation along with fibronectin production in airway constitutive cells

**DOI:** 10.1186/s12931-016-0342-7

**Published:** 2016-03-14

**Authors:** Taichi Shiobara, Kazuyuki Chibana, Taiji Watanabe, Ryo Arai, Yukiko Horigane, Yusuke Nakamura, Yumeko Hayashi, Yasuo Shimizu, Akihiro Takemasa, Yoshiki Ishii

**Affiliations:** Department of Pulmonary Medicine and Clinical Immunology, Dokkyo Medical University School of Medicine, 880 Kitakobayashi Mibumachi, Shimotsugagun, Tochigi 321-0293 Japan

**Keywords:** Bronchial asthma, Bronchial epithelial cells, Dipeptidyl peptidase-4, IL-13

## Abstract

**Background:**

Type 2 helper T-cell cytokines including IL-13 play a central role in the pathogenesis of bronchial asthma (BA). During the course of our research, our attention was drawn to dipeptidyl peptidase-4 (DPP4) as one of the molecules that were induced from bronchial epithelial cells (BECs) by IL-13 stimulation. DPP4 could become a new biomarker or therapeutic target. The aim of this study was to investigate the expression of DPP4 in the asthmatic airway, and its role in the pathophysiology of asthma.

**Methods:**

BECs were isolated from patients with inhaled corticosteroid-treated asthma (stBA) and inhaled corticosteroid-naïve asthma (snBA) using bronchoscopy.

*DPP4* mRNA expression in freshly isolated BECs and primary cultured BECs with or without IL-13 stimulation was investigated by microarray analysis and quantitative real-time PCR (qPCR). The distribution of DPP4 protein was determined by immunostaining of transbronchial lung biopsy specimens from asthma patients. The effect of recombinant human (rh) DPP4 on the proliferation of lung fibroblasts (HFL-1) and bronchial smooth muscle cells (BSMCs) was examined, as well as its effect on the production of fibronectin (FN).

**Results:**

*DPP4* mRNA was strongly expressed in freshly isolated BECs in snBA, and its expression was significantly enhanced by IL-13 stimulation. *DPP4* mRNA expression in BECs of snBA significantly correlated with exhaled nitric oxide. Biopsied tissues of the asthmatic airway revealed strong expression of DPP4 protein in BECs from snBA subjects. rhDPP4 stimulated the proliferation of HFL-1 and BSMCs, and it also enhanced production of FN from these airway cells.

**Conclusion:**

DPP4 may be involved in the pathologic features of asthmatic airway inflammation and cell proliferation and FN production.

**Electronic supplementary material:**

The online version of this article (doi:10.1186/s12931-016-0342-7) contains supplementary material, which is available to authorized users.

## Background

Chronic airway inflammation is a major pathologic feature of bronchial asthma, and type 2 helper T-cell (Th2) cytokines such as IL-4, IL-5 and IL-13 play important roles [1]. IL-13 stimulates bronchial epithelial cells (BECs) and causes the expression of various molecules involved in eosinophilic airway inflammation. Previously, we demonstrated that IL-13 induced the production of nitric oxide (NO) from BECs and acted on lung fibroblasts, in coordination with cysteinyl leukotrienes, resulting in airway inflammation and remodeling. Therefore, IL-13 has been widely recognized as playing an important role in the pathophysiology of asthma [2–5]. Furthermore, IL-13-induced periostin [6] can be measured in peripheral blood and is used as a companion marker for predicting the efficacy of anti-IL-13 antibodies in human asthma patients [7].

Recently, it was shown that DPP4 was induced in normal human BECs by IL-13 [8]. Another report demonstrated that the migration of CD4-positive T cells was decreased in a DPP4 (CD26)-deficient rat model of asthma [9].

DPP4 is a glycoprotein with a molecular weight of approximately 110,000, is composed of 766 amino acid residues [10], and is present on the cell membrane and in the blood as a soluble protein. Van der Velden investigated DPP4 expression in allergic asthma and normal controls, and showed that DPP4 was mainly expressed in blood vessels and mucosal glands and T cells [11]. DPP4 acts as a serine protease, which cleaves dipeptides from peptides containing proline or alanine residues. DPP4 degrades substrates such as incretin, neuropeptides, chemokines and substance P, mostly deactivating them [12]. DDP4 is also expressed as CD26 on the cell surface of immune cells. It is known to bind with molecules such as adenosine deaminase (ADA) and regulate intracellular signaling [13]. Recently, it was reported that DPP4 induced vascular smooth muscle growth [14], and it was reported that DPP4 inhibitors suppressed this action and contributed to the prevention of myocardial remodeling in a rat model [15]. In respiratory research, it was reported that eosinophilic airway inflammation was reduced in DPP4-deficient rats [16]. However, the localization of DPP4 in the airway in non-treated asthma patients and the direct effects of DPP4 on the pathophysiology of asthma are not fully understood.

In this study, we used DNA microarray analysis to comprehensively screen for candidate genes that were differentially expressed between inhaled corticosteroid-naïve asthma (snBA) and inhaled corticosteroid (ICS)-treated asthma (stBA) and showed enhanced expression in the freshly isolated BECs. We also performed microarray analysis using IL-13-stimulated and un-stimulated primary cultured BECs. Based on the results of these analyses, our attention became focused on DPP4. There are numerous reports about genes induced by IL-13 [8, 17, 18], but little information is available on DPP4. Our attention was drawn to DPP4, which is expressed in BECs after IL-13 stimulation and DPP4 expression is significantly enhanced in the BECs of snBA patients. We investigated the correlation between DPP4 and exhaled nitric oxide (eNO), and analyzed the localization of DPP4 in BECs, using airway samples from patients with stBA and snBA. We also examined the effects of recombinant human (rh) DPP4 on fibronectin (FN) expression and production using lung fibroblasts and bronchial smooth muscle cells (BSMCs).

## Methods

### Study population

We conducted a retrospective study of 31 asthmatic patients, comprising 13 with stBA and 18 with snBA, who visited the Department of Pulmonary Medicine and Clinical Immunology of Dokkyo University Hospital from June 2009 to March 2014. For the analysis of the expression of *DPP4* mRNA, bronchial brushings were performed in these patients (Table 1), including the 7 cases whose cells were used for the microarray analysis (Table 2). Transbronchial lung biopsy (TBLB) and end-bronchial biopsy (EBB) were performed in patients whenever possible.

**Table 1:** Characteristics of all subjects of this study

	M : F	Age	%FEV_1_	FEV_1_/FVC	eNO(ppb)	N : E : C
stBA	10 : 3	51 ± 4	88 ± 6	73 ± 4	45 ± 5	3 : 8 : 2
snBA	13 : 5	48 ± 4	83 ± 6	72 ± 3	129 ± 22*	6 : 10 : 2

Data are mean ± SEM. *p < 0.01 vs stBA

**Table 2:** Characteristics of subjects whose cells were analyzed by DNA microarray

	Number	Age	%FEV_1_	FEV_1_/FVC	eNO(ppb)	N : E : C
stBA	4	47 ± 6	96 ± 6	79 ± 5	58 ± 10	1 : 1: 2
snBA	3	48 ± 4	91 ± 7	72 ± 1	123 ± 25*	1 : 2 : 0

Data are mean ± SEM. *p < 0.05 vs stBA

Definition of abbreviations: N; never smoker, E; ex-smoker, C; current smoker

All subjects met the American Thoracic Society criteria for asthma. Subjects had an FEV1 greater than 80 % of the predicted value and their FEV1/FVC was greater than 70 %. Subjects with stBA were regularly treated with inhaled corticosteroid. Subjects with snBA visiting our hospital for the first time had slight symptoms such as a continuous cough with wheezing and nighttime dyspnea, and they had never been treated with inhaled or oral corticosteroids. Written informed consent was obtained from participants. This study was approved by the Ethics Committee of Dokkyo University School of Medicine (hop-m22095).

### Bronchoscopy with bronchial epithelial cell brushing

Bronchial brushings were performed with a standard, sterile, single-sheathed nylon cytology brush (Olympus T-260; Olympus, Tokyo, Japan). A total of 4 sets of 10 brushings were performed in the distal airways. The distal BECs were obtained from the airway situated about 1 cm away from the pleura—equivalent to the airway of the 10^th^ to 15^th^ branch of Wiebel’s model—with a diameter of less than 2 mm, which are the so-called distal airways [19]. The mean cell counts of BECs were 4.4 ± 0.6 × 10^5^ for distal BECs, and 4.9 ± 1.1 × 10^5^ for proximal BECs, with purity 90 % and viability 80 %, respectively. Whenever possible, biopsies were performed to obtain samples for immunohistochemistry.

### Microarray analysis

Total RNA was extracted from freshly isolated BECs and primary cultured BECs using Trizol (Invitrogen, Carlsbad, CA) and the RNeasy Micro kit (QIAGEN, GmBH, Germany). RNA was quantified by spectrometry using NanoDrop ND1000, (NanoDrop Technologies, Wilmington, DE) and the quality of the RNA was confirmed using an Agilent 2100 Bioanalyzer (Agilent Technologies, Santa Clara, CA). RNA amplification products were converted into cDNA. Microarray was conducted in three of snBA and four of stBA and also ALI-cultured with or without IL-13 treated BECs (*N* = 3 respectively). Arrays were scanned in an Agilent Microarray Scanner (Agilent Technologies) according to the manufacturer’s protocol and data were extracted using Agilent Feature Extraction Software 10.7.3.1 following the Agilent protocol. Data analysis was carried out by using GeneSpring GX 12.0 software (Agilent Technologies). Sample quality control was carried out with GeneSpring® software and, based on Flag information, unreliable samples and entities were excluded. In addition, data was normalized with a percentile shift to the 75 % percentile, and the errors between experiments were corrected by aligning the baseline with the median of all controls. We have presented the 3D PCA score for microarray analysis performed on freshly isolated BECs and ALI-cultured BECs. Data is shown in Additional file 1: Figure S1.

### Cell culture

Freshly isolated BECs were seeded into 60 mm tissue-culture dishes coated with rat-tail type I collagen (BD Discovery Labware, Bedford, MA). When the BECs reached 80 % confluence, cells were passaged and seeded onto collagen-coated polyester 12-well Transwell inserts with BEBM/DMEM. When the cell layer was 100 % confluent in the transwells, the culture method was shifted to the air-liquid interface (ALI) method by removing all of the apical medium. The ALI state was maintained for 10 days, as previous studies have demonstrated that this time is required for mucociliary differentiation [2, 20].

Human fetal lung fibroblasts (HFL-1; lung, diploid, human, passage 3–7 cells) were obtained from the American Type Culture Collection (Manassas, VA). Human BSMCs (C-12561; passages 3–7) were obtained from TaKaRa (Shiga, Japan). HFL-1 or BSMCs were seeded into 24-well tissue culture plates at a density of 4 × 10^4^ cells/well and were cultured in their respective conditioned medium until confluence. The medium was then replaced with 0.5 % FBS conditioned medium and cells were stimulated with rhDPP4 (Abnova P3467, CA) at a concentration ranging from 0 to 500 ng/ml for 24 to 96 h.

For analysis of cell proliferation**,** HFL-1 and BSMCs were seeded into 96-well tissue culture plates at a density of 1 × 10^4^ cells/well and were cultured in conditioned medium until sub-confluence. The cells were then washed twice and the medium was replaced with serum-free conditioned medium. After culture for 24 h, this medium was replaced with conditioned medium containing 0.5 % FBS and the cells were incubated for another 24 h in the presence or absence of various (0 to 500 ng/ml) concentrations of rhDPP4. All cells were cultured at 37 °C in a 5 % CO_2_ humidified incubator in conditioned medium.

### Quantitative real-time PCR

The expression of *DPP4* mRNA by BECs and the expression of *FN* mRNA by HFL-1 and BSMCs were determined by reverse transcription (RT), followed by real-time quantitative PCR as described previously [4, 5]. First-strand cDNA was synthesized using the PrimeScript RT reagent Kit (TaKaRa) with both oligo(dT) primers and random hexamers.

Reverse transcription was performed with a TaKaRa PCR Thermal Cycler MP (TP3000). The following are the sequences of the primers used for amplification of *DPP4*, *FN* and *GAPDH*:

- *DPP4*: forward primer, GCACGGCAACACATTGAA;
- reverse primer, TGAGGTTCTGAAGGCCTAAATC;
- *FN*: forward primer, CTTTGGTGCAGCACAACTTC;
- reverse primer, CCTCCTCGAGTCTGAACCAA
- *GAPDH*: forward primer, GCACCGTCAAGGCTGAGAAC;
- reverse primer, TGGTGAAGACGCCAGTGGA.

DNA was amplified for 40 cycles of denaturation for 5 s at 95 °C and annealing for 30 s at 60 °C, using the TaKaRa Thermal Cycler Dice (TP900). The PCR assays were performed and analyzed using the Thermal Cycler Dice Real Time System version 4.2 (TaKaRa). The specificity of reactions was determined by melting curve analysis. The relative expression of each gene of interest and of *GAPDH* were calculated using the ΔΔCt method.

### Quantification of DPP4 and fibronectin by ELISA

Cell culture supernatant was collected from ALI cultured BECs, BSMC and HFL-1 and were stored at -80 °C. DPP4 (R&D Systems, Minneapolis, Minn) or fibronectin (TakaraBio Inc, Shiga, Japan) were used in standardized sandwich ELISAs, according to the manufacturer’s protocol.

### Correlations between eNO and DPP4 expression

We also measured eNO before bronchoscopy at a flow rate of 50 ml/s using the nitric oxide analyzer (NOA) 280i® (Sievers, CO). Correlations between eNO and *DPP4* mRNA expression in distal BECs from asthma subjects were analyzed.

### Immunohistochemistry

Transbronchial lung biopsy specimens from 5 snBA patients and 5 stBA patients were fixed in formalin. Serial 4-μm sections were immunostained using a rabbit polyclonal antibody against DPP4 (1:500) (Abcam, MA). Immunohistochemistry was performed using the Dako EnVisionTM FLEX Mini Kit High pH detection system. DPP4 expression in the epithelial layer was scored semi-quantitatively on a scale of 1 to 4 (1 = negative, 2 = weak, 3 = moderate, 4 = strong) by four independent observers, who were unaware of the sample's identity. The final scores of DPP4 expression were the mean value of the four observations and compared between snBA and stBA.

### Cell proliferation stimulated by rhDPP4

HFL-1 and BSMC proliferation was evaluated on the basis of DNA synthesis that was assessed by measuring 5-bromo-29-deoxyuridine (BrdU) incorporation with an ELISA kit (Roche), as previously described [5]. This assay was performed according to the manufacturer’s instructions.

### Statistical analysis

Statistical analysis was performed using the Wilcoxon rank sum test (Mann–Whitney *U* test) to compare between control and each stimulated response. For all comparisons, P-values < 0.05 were considered significant. The statistical software used was the JMP version 10 (SAS Institute, Cary, NC).

## Results

### DNA microarray analysis of BECs from asthma patients

We performed DNA microarray analysis for identifying differences in gene expression between snBA and stBA. Table 1 shows the characteristics of subjects in this study. *DPP4* mRNA expression in BECs were analyzed retrospectively in 31 subjects (stBA, *n* = 13; snBA, *n* = 18). Four of the subjects were current smokers and 18 subjects had a history of smoking. None of these patients had COPD; their FEV1/FVC was over 70 % and they had no low attenuation area on CT. For the snBA patients, the average eNO level was 128.8 ppb and this value was significantly higher than that of stBA patients (44.6 ppb). Table 2 shows the characteristics of the subjects whose samples were used for DNA microarray analysis. In the snBA group, the average age was 49 years old, the average FEV1/FVC was 72 % and the average eNO level was 123 ppb. In the stBA group, the average age was 47 years old, the average FEV1/FVC was 78.6 % and the average eNO level was 58 ppb. All cases were men.

Genes expressed in freshly isolated distal BECs from snBA and stBA patients in the stable phase were compared using microarray analysis, and 433 genes whose expression was enhanced greater than 2-fold in snBA were extracted. In addition, using primary cultured distal BECs derived from snBA patients (*n* = 3), which were cultured using an ALI method, we examined the gene expression of cells that had been cultured for 10 days in media with or without IL-13 (10 ng/mL), and extracted 692 genes whose expression was enhanced greater than 2-fold as a result of IL-13 stimulation.

**Fig. 1:**
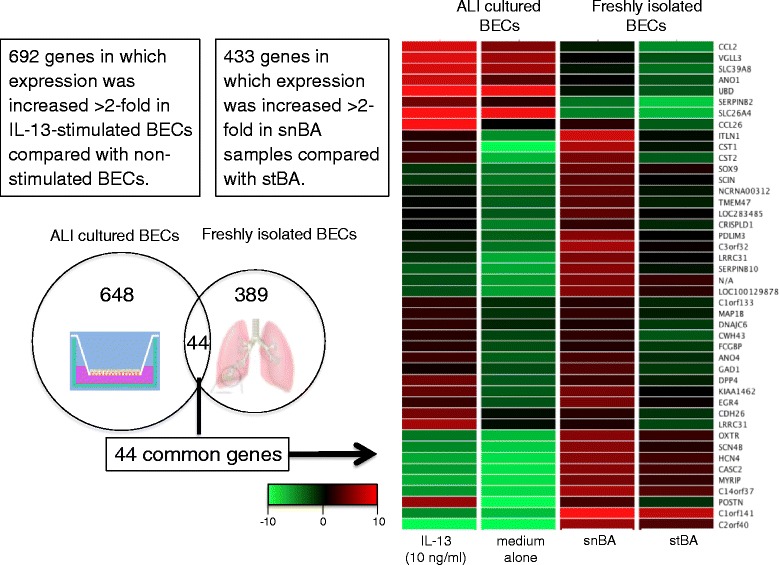
Results of DNA microarray analysis. In the ALI group, there were 692 genes whose expressions were enhanced more than 2-fold by IL-13 (10 ng/ml) stimulation. In the freshly isolated BEC group, there were 433 genes whose expressions were enhanced more than 2-fold in snBA samples. The 44 genes common to both groups were extracted from the Venn diagram and displayed together with a heatmap

There were 44 genes with greater than 2-fold enhanced expressions that were common to both ALI-cultured and freshly isolated BECs (Fig. 1). The 44 genes whose expression was induced in these BECs by IL-13 and that had decreased expression as a result of therapeutic intervention in asthma patients. Among the genes, DPP4 attracted our attention. DPP4 was 2.24-fold increased in snBA compare to stBA of freshly isolated BECs. Also, DPP4 was increased 6.75-fold increased in IL-13 treated group of ALI-cultured BECs. DPP4 is a one the gene that variable in untreated asthma and IL-13 stimulation.

### *DPP4* mRNA expression in freshly isolated distal BECs and correlation with eNO

**Fig. 2:**
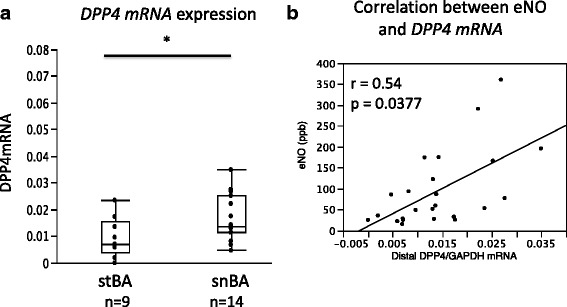
*DPP4* mRNA expression in freshly isolated BECs from asthma patients by qPCR. *DPP4* mRNA was significantly enhanced in distal BECs in snBA. ^*^ *P* < .05 compared with stBA.  **b**. DPP4 mRNA expression in distal BECs significantly correlated with eNO (*r*= 0.54, *p*=0.0377)

The expression of *DPP4* mRNA measured by qPCR in freshly isolated BECs obtained from asthma patients by bronchial brushing was significantly higher in snBA compared with stBA, in distal airway samples. No significant differences in DPP4 mRNA and protein were found in primary cultures between snBA and stBA patients (Fig. 2a). Significant correlations were seen between *DPP4* mRNA expression in the distal airways and eNO in asthma patients (Fig. 2b: *r* = 0.54, *p* = 0.0377). DPP4 mRNA also showed significant correlation with *iNOS* mRNA. This is mentioned in the Discussion and illustrations are provided in Additional file 2: Figure S2.

### DPP4 expression and production induced by IL-13 in cultured BECs in vitro

**Fig. 3:**
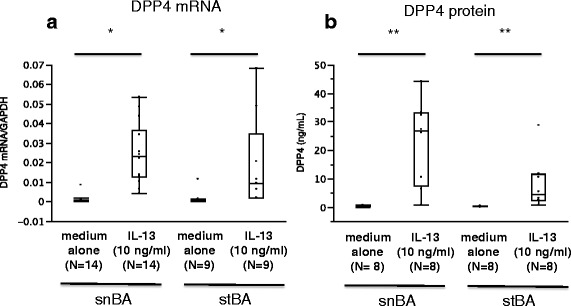
*DPP4* mRNA and protein induced by IL-13 in ALI-cultured BECs. **a** In primary cultured BECs derived from both snBA and stBA samples, DPP4 mRNA expression was enhanced by IL-13 (10 ng/ml) stimulation. ^*^ *P* < .001, compared with medium alone. **b** The production of DPP4 protein was similarly enhanced by IL-13 (10 ng/ml) stimulation, in cells from both snBA and stBA samples. ^**^ *P* < .01, compared with medium alone

Primary cultured BECs isolated from snBA and stBA patients were cultured by the ALI method and *DPP4* mRNA expression and protein production with or without IL-13 stimulation (10 ng/ml) were compared. *DPP4* mRNA expression was significantly enhanced with IL-13 stimulation, in BECs from both snBA patients and stBA patients. DPP4 protein production by measuring by ELISA was also significantly enhanced by IL-13 stimulation (Fig. 3). There are no significant differences between snBA and stBA in DPP4 mRNA and protein.

### Immunohistochemistry

**Fig. 4:**
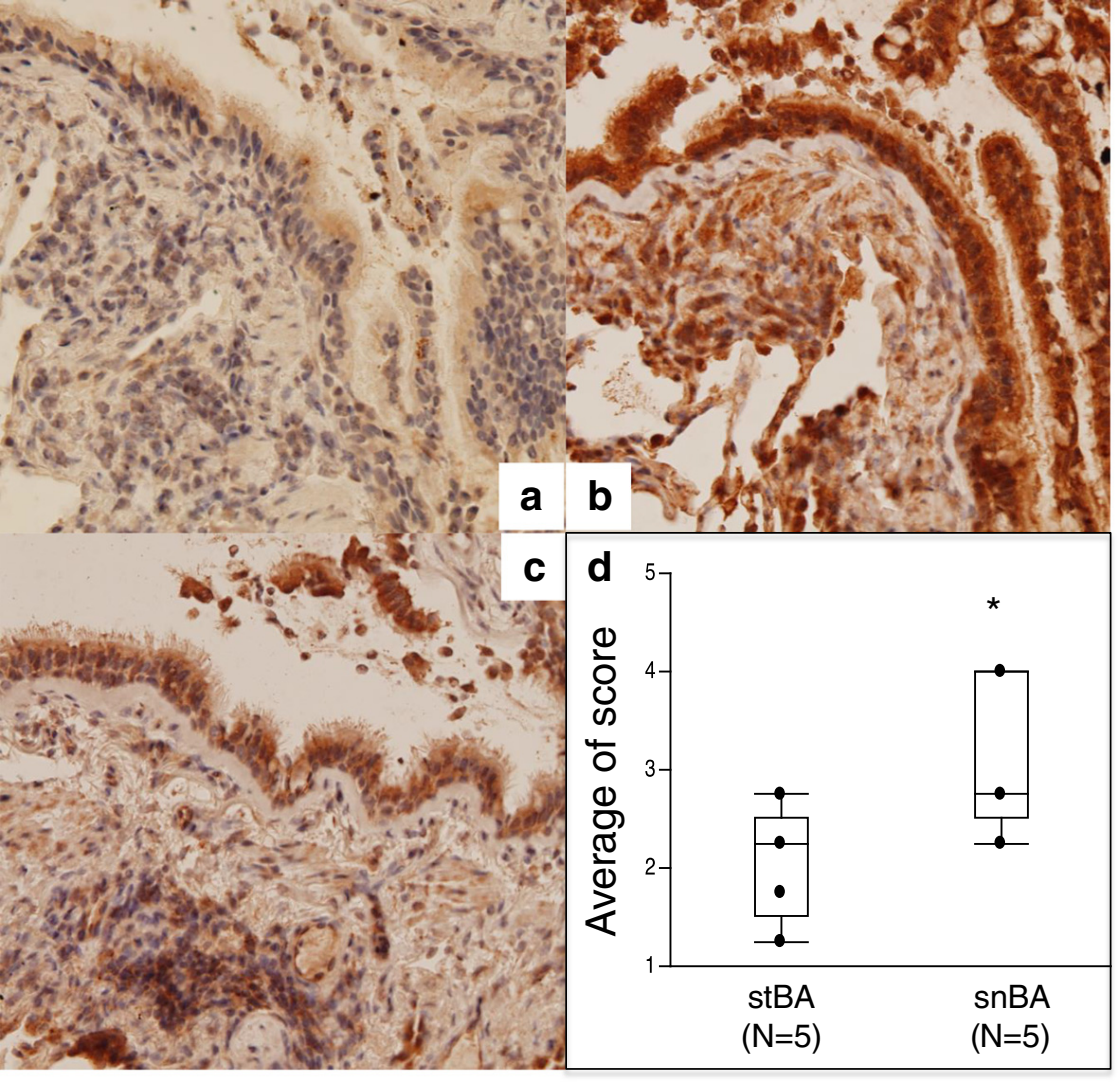
Immunohistochemistry of airway tissue. Distal BECs of snBA samples were stained with **a** IgG isotype (×200) or **b** anti-DPP4 antibody. DPP4 expression can be seen in inflammatory cells, and staining is strongest in the BECs. A sample from a stBA patient after 8 months of ICS treatment (**c**). Semi-quantitative analysis of DPP4 intensity scored on a 4-point scale (**d**). DPP4 protein expression is significantly higher in snBA (*N* = 5, respectively). ^*^ *P* < .05, compared with stBA

The distribution of DPP4 protein expression in the distal airway tissues obtained from asthma patients by biopsy using bronchoscopy was investigated by immunohistochemistry; representative examples are shown in Fig. 4. Comparison of samples from asthma patients that were immunostained with control isotype IgG (Fig. 4a) or with an anti-DPP4 antibody (Fig. 4b, representative snBA patient) indicated that the DPP4 protein was strongly expressed in BECs and in inflammatory cells such as eosinophils, macrophages and lymphocytes. This expression was reduced in the representative stBA patient (Fig. 4c). Figure 4d shows the scores of DPP4 intensity that were determined on a 4-point scale. Comparison of the expression of DPP4 in bronchial biopsies of stBA and snBA patients (*N* = 5, respectively) revealed significantly reduced DPP4 intensity in the BECs of stBA (*p* < 0.05).We explored whether steroids directly suppress DPP4 expression in vitro. DPP4 induced by IL-13 was significantly inhibited by mometasone furoate (MF). This is shown in Additional file 3: Figure S3. We also investigated the expression of DPP4 in asthma patients and non-asthma patients who had undergone surgery for lung cancer. As expected, the asthma group showed more staining than the non-asthma group. Representative photos are shown in Additional file 4: Figure S4.

### Proliferation of HFL-1 and BSMCs stimulated by rhDPP4

**Fig. 5:**
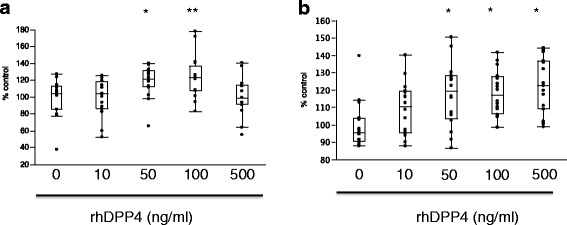
Assay of the effect of rhDPP4 on the proliferation of BSMCs and HFL-1 cells. **a** Proliferative effect of rhDPP4 on BSMCs. Significant proliferation was seen with rhDPP4 at 50 and 100 ng/ml. But a proliferative effect was not observed at 500 ng/ml. *P < .005 and **P < .05, compared with no rhDPP4. **b** In HFL-1, a concentration-dependent proliferative effect was observed at rhDPP4 concentrations of 50 ng/ml and above. *P < .01, compared with no rhDPP4

To investigate the involvement of DPP4 in airway remodeling in asthma patients, we examined the proliferative effect of rhDPP4 on HFL-1 and BSMCs. At 24 h, a proliferative effect on BSMCs was observed after rhDPP4 stimulation (Fig. 5). The concentration of rhDPP4 that stimulated the highest growth was 100 ng/ml for BSMCs and 500 ng/ml for HFL-1.

### FN mRNA and protein expression in BSMCs and HFL-1 stimulated by rhDPP4

**Fig. 6:**
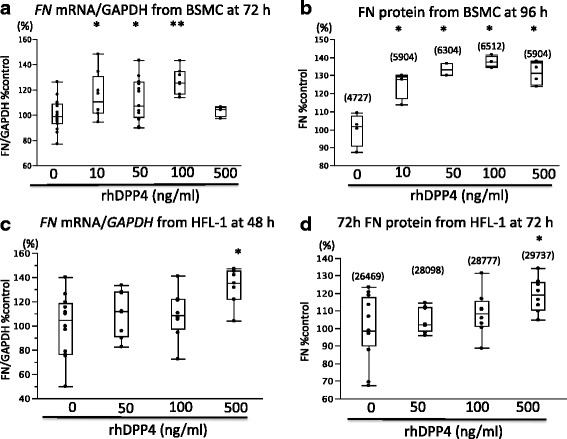
*FN* mRNA expression and protein production in BSMCs and HFL-1 stimulated by rhDPP4. **a** rhDPP4-induced *FN* mRNA expression in BSMCs at 72 h. ^*^ *P* < .05 and ^**^ *P* < .001, compared with no rhDPP4. **b** rhDPP4-induced FN protein production in BSMCs at 96 h. ^*^ *P* < .05, compared with no rhDPP4. The actual mean concentrations of FN protein (ng/mL) in BSMCs were shown in **b** as control = 4726.8, DPP4 (10 ng/mL) = 5903.5, DPP4 (50 ng/mL) = 6304.46, DPP4 (100 ng/mL) = 6512.1, and DPP4 (500 ng/mL) = 5903.5. **c** rhDPP4-induced FN mRNA expression in HFL-1 at 48 h. ^*^ *P* < .05, compared with no rhDPP4. **d**. rhDPP4-induced protein production in HFL-1 at 72 h. ^*^ *P* < .05, compared with no rhDPP4. The actual mean concentrations of FN protein (ng/mL) in HFL-1 were shown in **b** as control = 26469, DPP4 (50 ng/mL) = 28098, DPP4 (100 ng/mL) = 28777, and DPP4 (500 ng/mL) = 29737

To examine the direct effect of DPP4, we tested rhDPP4-induced FN production in BSMCs (Fig. 6a and b) and HFL-1 (Fig. 6c and d). rhDPP4-stimulated expression of *FN* mRNA in BSMCs and HFL-1 was investigated by qPCR and FN protein concentrations were measured by ELISA. With regard to FN, rhDPP4 stimulation enhanced the expression of *FN* mRNA and FN protein production in a concentration-dependent manner. The results were variable, hence the data were expressed as percentage changes from baseline. We also described actual number of mean protein concentration in the Fig. 6c and d.

## Discussion

There were two main findings in this study. First, DPP4 expressions were enhanced in the BECs of asthma patients, especially in snBA, and also in BECs stimulated by IL-13. Second, rhDPP4 stimulates the production of FN and has proliferative effects on HFL-1 and BSMCs.

With regards to the first finding, we thought that previous investigations [2–7] of molecules that are involved in the pathophysiology of asthma through IL-13 stimulation are important not only for the diagnosis of asthma but also for identifying markers that predict disease activity and response to treatment. However, the role of DPP4 in the pathogenesis of asthma is still unclear, and revealing this role could potentially lead to greater understanding of the pathophysiology and treatments of asthma.

In the field of asthma research, one study revealed that *DPP4* mRNA expression was induced by IL-13 stimulation of normal human BECs by microarray analysis [8] but it did not examined DPP4 in detail. In our study, a thorough investigation using microarray analysis, qPCR and ELISA was conducted and we found that *DPP4* expression and production were induced in both BECs from asthma patients and in cultured BECs stimulated by IL-13. Significant correlations were seen between *DPP4* mRNA expression in the distal airways and eNO in asthma patients. Interestingly, *DPP4* mRNA expression is correlated with *iNOS* mRNA both in distal and proximal airways. (*R* = 0.48, *p* = 0.0176 and *R* = 0.59, *p* = 0.0033, respectively.) Data is shown in Additional file 4: Figure S4.

In an animal model of asthma using DPP4 (CD26)-deficient rats, it was reported that CD4-positive T-cell migration decreased even with IL-13 stimulation [9, 21]. In previous studies, it has often been pointed out that DPP4 is linked to airway inflammation in animal models of asthma. In our study, snBA patients who underwent bronchoscopy had a high mean exhaled NO level of 128.8 ppb, which reflects airway inflammation, and stBA patients undergoing ICS therapy had a relatively low mean exhaled NO of 44.6 ppb. *DPP4* mRNA levels measured directly using snBA samples were higher than in distal airway samples from stBA patients. We also observed by immunostaining that DPP4 expression was enhanced in the airways, especially in BECs in the setting of snBA. These data suggesting that DPP4 protein expression could decrease in these patients following ICS therapy. This is not inconsistent with the finding of reduction of airway inflammation in DPP4-deficient rats [9]. Previously, van der Velden et al reported that the expression of DPP4 and its enzyme activity were seen not in epithelial cells mainly but in blood vessels, mucosal glands and T-cells. They found no difference in DPP4 expression between allergic asthma and healthy subjects [11]. The discrepancy between their study and ours may be due to the fact that allergic asthma subjects had not taken oral or inhaled corticosteroid in the 1 month prior to their study, but our snBA patients had never been treated with oral or inhaled corticosteroids. The antibody used in the previous report was a monoclonal antibody, whereas we used a polyclonal antibody, thus the recognized epitopes and sensitivities could also have been different between the studies. Differences may also exist between their immunostaining methods and ours. For example, we used a high-sensitivity visualization system (Dako EnVision FLEX Mini Kit) because DPP4 stains well after using this system in our study. Using this system may have influenced the results.

Next, we will discuss about the second finding of this study: cellular proliferation and FN production by rhDPP4-stimulated BSMCs and HFL-1. It has been previously reported that DPP4 has three main functions [22]. The first known function is the immunostimulatory action of DPP4/CD26, which is primarily expressed on the surface of lymphocytes. The best known immunostimulatory activity of DPP4 is its contribution to T lymphocyte activation [23]. T cell activation occurs when antigen-presenting cells (APC) present antigens using the major histocompatibility complex to the T-cell receptor on T lymphocytes. During this process, caveolin-1 on APC binds with DPP4/CD26 on the surface of T cells, thereby contributing to T-cell activation through a co-stimulatory effect [24]. The second function of DPP4 is adhesion on the surface of cells, which is mediated by collagen and FN. The third function of DPP4 is its activity as a serine protease. Specifically, DPP4 is released in a soluble form into the blood from the surface of cells of various tissues, and acting as a serine protease, it degrades incretin, gastrointestinal hormones, neuropeptides and chemokines, deactivating these molecules in many cases [12, 25]. This action leads to the exacerbation of blood glucose control in diabetes mellitus. Currently, DPP4 inhibitors are being used to treat diabetes mellitus and it is a useful therapeutic approach. In the field of cardiology, it has been reported that DPP4 induces vascular smooth muscle growth [26] and that DPP4 inhibitors suppress this action and contribute to the prevention of myocardial remodeling in mice and human [15, 27, 28].

Accordingly, we hypothesized that DPP4 would affect airway remodeling, and we investigated the effects of DPP4 on BSMCs and HFL-1.

In in vitro experiments, we found that rhDPP4 had a proliferative effect on HFL-1 and BSMCs (Fig. 5), and these findings were similar to those of a previous study reporting that DPP4 had a proliferative effect on vascular smooth muscle cells [14].

We also found that rhDPP4 induced *FN* mRNA expression and protein production in BSMCs and HFL-1 in a concentration-dependent manner (Fig. 6).

The above results suggest that rhDPP4 has a direct proliferative effect on BSMCs and HFL-1 and induces the production of extracellular matrix in asthma and could be involved in airway remodeling.

Currently, the pathways and receptors of DPP4 are unclear. However, the fact that DPP4 has properties as a serine protease suggests that, similar to thrombin, its cell proliferation and FN production effects might be mediated by receptors such as protease-activated receptor 2 (PAR2) [26] and insulin-like growth factor II receptor (IGF2R) [29]. Soluble DPP4 directly activates the MAPK and NF-κB signaling cascade involving PAR2 and resulting in the induction of inflammation and proliferation of human vascular smooth muscle cells [26]. In addition, DPP-4 dose-dependently increased reactive oxygen species (ROS) generation and advanced glycation end products (AGEs) and receptor (RAGE) gene expression in endothelial cells, which were prevented by anti-IGFR2 antibody blocked the ROS generation in DPP-4-exposed endothelial cells [29]. These reports suggested that DPP4 act not only as an enzyme, but also activated some receptors and signal pathways.

This study has some limitations because it was retrospective in design. First, the microarray analysis performed in this study could not compare gene expression between asthma patients and healthy individuals, but rather, it compared gene expression between snBA and stBA, and all patients were men. Ideally, a comparison of gene expression in the BECs of snBA patients and healthy individuals should be conducted to identify genes with enhanced expression in asthma. However, it is difficult to perform bronchoscopies in healthy individuals, which is one of the limitations of our study. Second, *DPP4* mRNA and protein levels were examined in a limited number of patients in our study. Bronchoscopies performed in asthma patients carry a risk of inducing an attack. As a result, the number of patients subjected to both microarray analysis and qPCR was limited.

Future in vitro studies will need to demonstrate whether DPP4 inhibitors or glucocorticoids prevent FN expression and production from lung fibroblasts and bronchial smooth muscle cells. Also, in vivo studies on the effect of DPP4 or DPP4 inhibitors using animal models of asthma will be needed. An investigation of the receptors and signaling pathways involved in IL-13-mediated enhancement of DPP4 expression should also be conducted.

## Conclusions

We demonstrated that DPP4 is expressed in BECs in human asthma and is induced by IL-13. Our findings also suggest that DPP4 is involved in promoting the growth of BSMCs and lung fibroblasts, in enhancing the production of FN.

## Additional files

Additional file 1: 3D PCA score for microarray analyses performed on freshly isolated BECs and ALI-cultured BECs. The stBA and snBA group appear similar because one case analyzed before and after treatment was included; however, overall they seemed to be divisible into two groups. The control and IL-13 groups were clearly divisible into two groups. (PPTX 123 kb) Additional file 2: DPP4 mRNA showed significant correlation with iNOS mRNA in the freshly isolated BECs from snBA. (PPTX 1447 kb) Additional file 3: Representative DPP4 immunostaining with surgical resected lung. The asthma group showed more staining than the non-asthma group. (PPTX 101 kb) Additional file 4: DPP4 mRNA and protein induced by IL-13 was significantly inhibited by mometasone furoate (MF). (PPTX 129 kb)

## Abbreviations

BECs: bronchial epithelial cells
DPP4: dipeptidyl peptidase-4
EBB: endobronchial biopsy
eNO: exhaled nitric oxide
FN: fibronection
rhDPP4: recombinant human DPP4 protein
snBA: inhaled corticosteroid-naïve bronchial asthma
stBA: inhaled corticosteroid (ICS)-treated bronchial asthma
TBLB: transbronchial lung biopsy
