# Supplementary material for: Dipeptidyl peptidase-4 is highly expressed in bronchial epithelial cells of untreated asthma and it increases cell proliferation along with fibronectin production in airway constitutive cells
Source: Respir Res. 2016 Mar 14;17:28. doi: 10.1186/s12931-016-0342-7 (PMC4791890; doi:10.1186/s12931-016-0342-7)
Supplement: Additional file 1: — 3D PCA score for microarray analyses performed on freshly isolated BECs and ALI-cultured BECs. The stBA and snBA group appear similar because one case analyzed before and after treatment was included; however, overall they seemed to be divisible into two groups. The control and IL-13 groups were clearly divisible into two groups. (PPTX 123 kb) [file 12931_2016_342_MOESM1_ESM.pptx]

## Slide 1
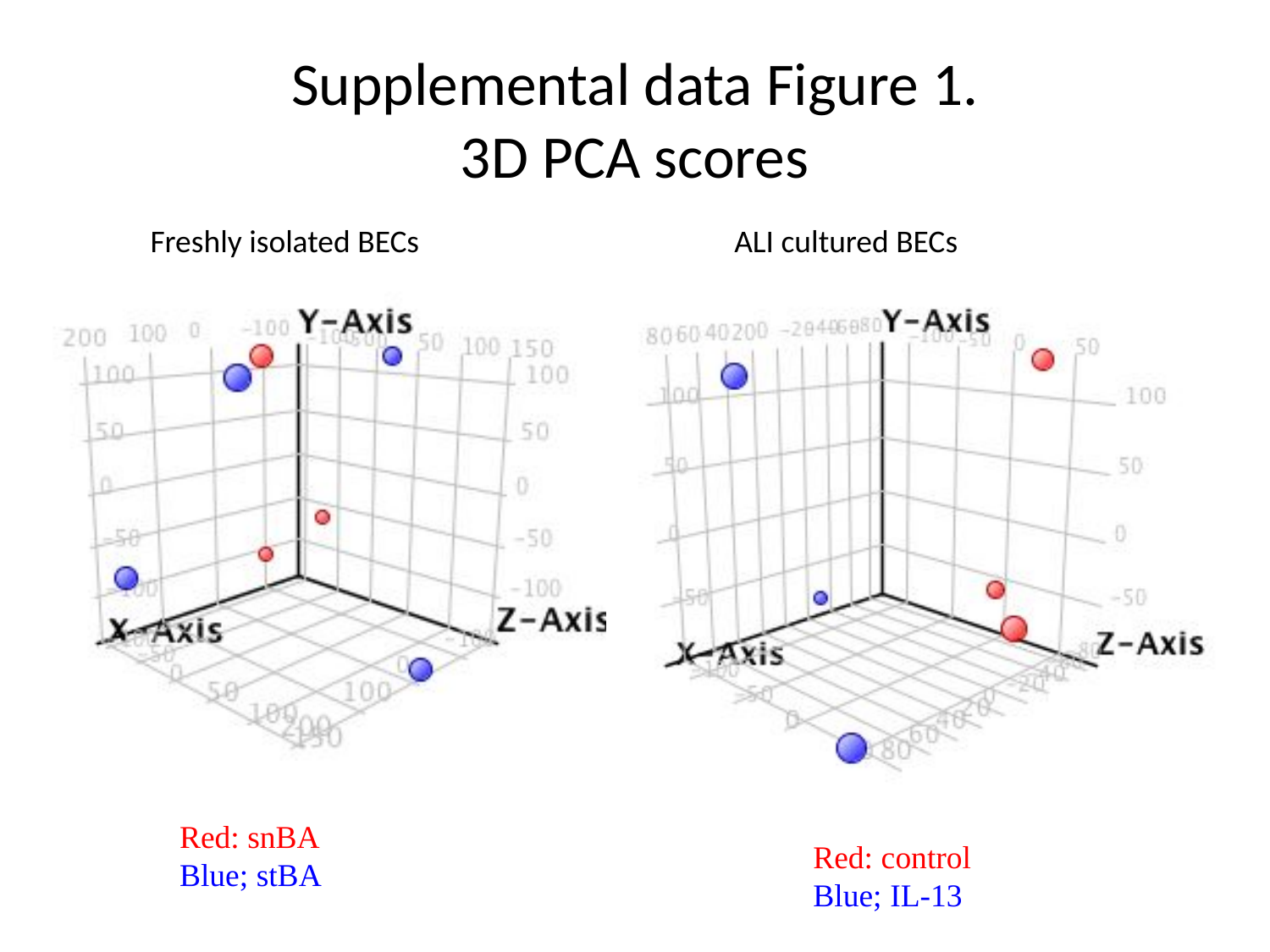

# Supplemental data Figure 1. 3D PCA scores
Freshly isolated BECs
ALI cultured BECs
Red: snBA
Blue; stBA
Red: control
Blue; IL-13
