# Supplementary material for: Dipeptidyl peptidase-4 is highly expressed in bronchial epithelial cells of untreated asthma and it increases cell proliferation along with fibronectin production in airway constitutive cells
Source: Respir Res. 2016 Mar 14;17:28. doi: 10.1186/s12931-016-0342-7 (PMC4791890; doi:10.1186/s12931-016-0342-7)
Supplement: Additional file 2: — DPP4 mRNA showed significant correlation with iNOS mRNA in the freshly isolated BECs from snBA. (PPTX 1447 kb) [file 12931_2016_342_MOESM2_ESM.pptx]

## Slide 1
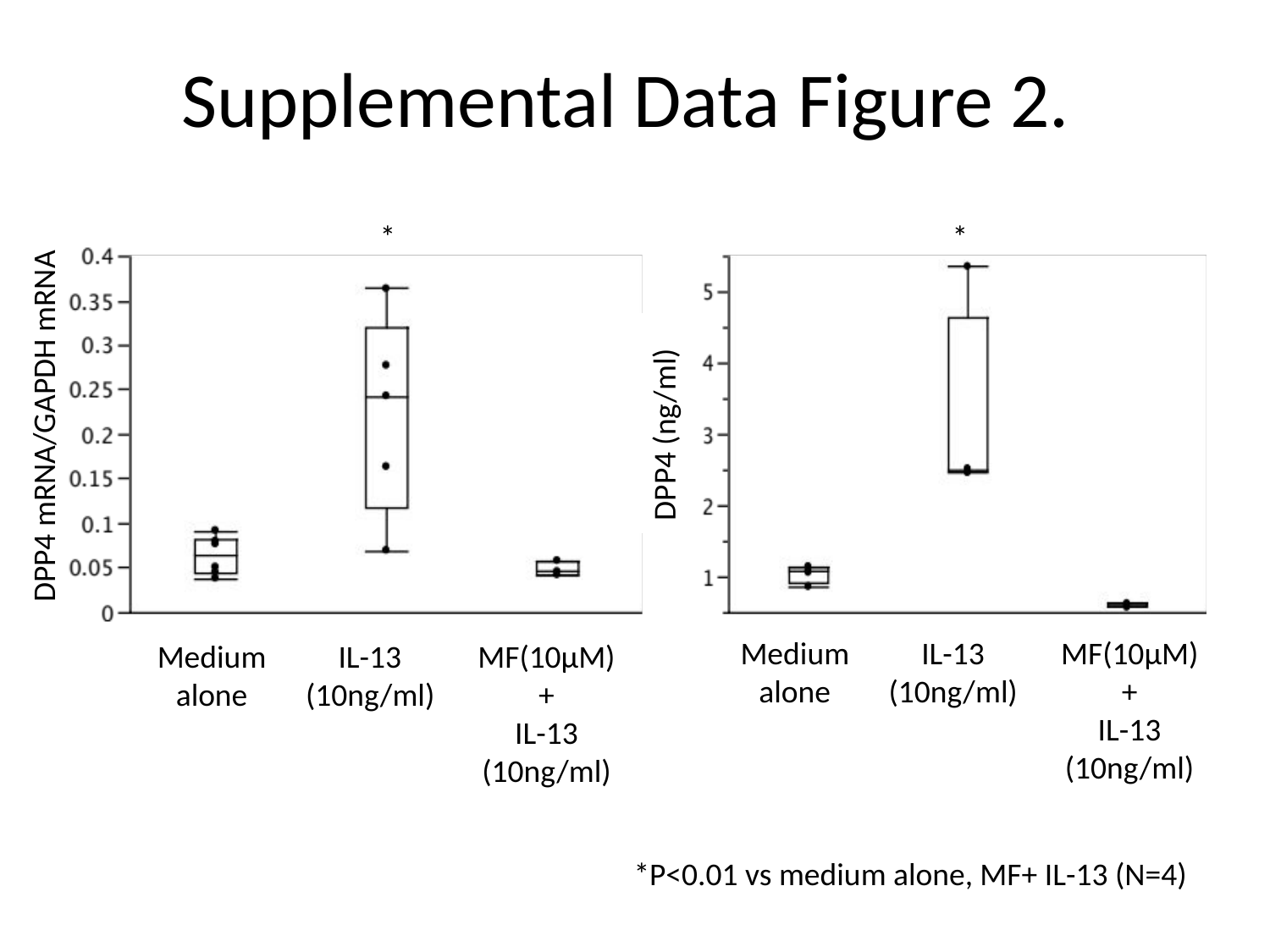

# Supplemental Data Figure 2.
*
*
DPP4 (ng/ml)
DPP4 mRNA/GAPDH mRNA
Medium alone
IL-13
(10ng/ml)
MF(10µM)
+
IL-13
(10ng/ml)
Medium alone
IL-13
(10ng/ml)
MF(10µM)
+
IL-13
(10ng/ml)
*P<0.01 vs medium alone, MF+ IL-13 (N=4)
