# Supplementary figures and images for: Dipeptidyl peptidase-4 is highly expressed in bronchial epithelial cells of untreated asthma and it increases cell proliferation along with fibronectin production in airway constitutive cells
Source: Respir Res. 2016 Mar 14;17:28. doi: 10.1186/s12931-016-0342-7 (PMC4791890; doi:10.1186/s12931-016-0342-7)

## Slide 1
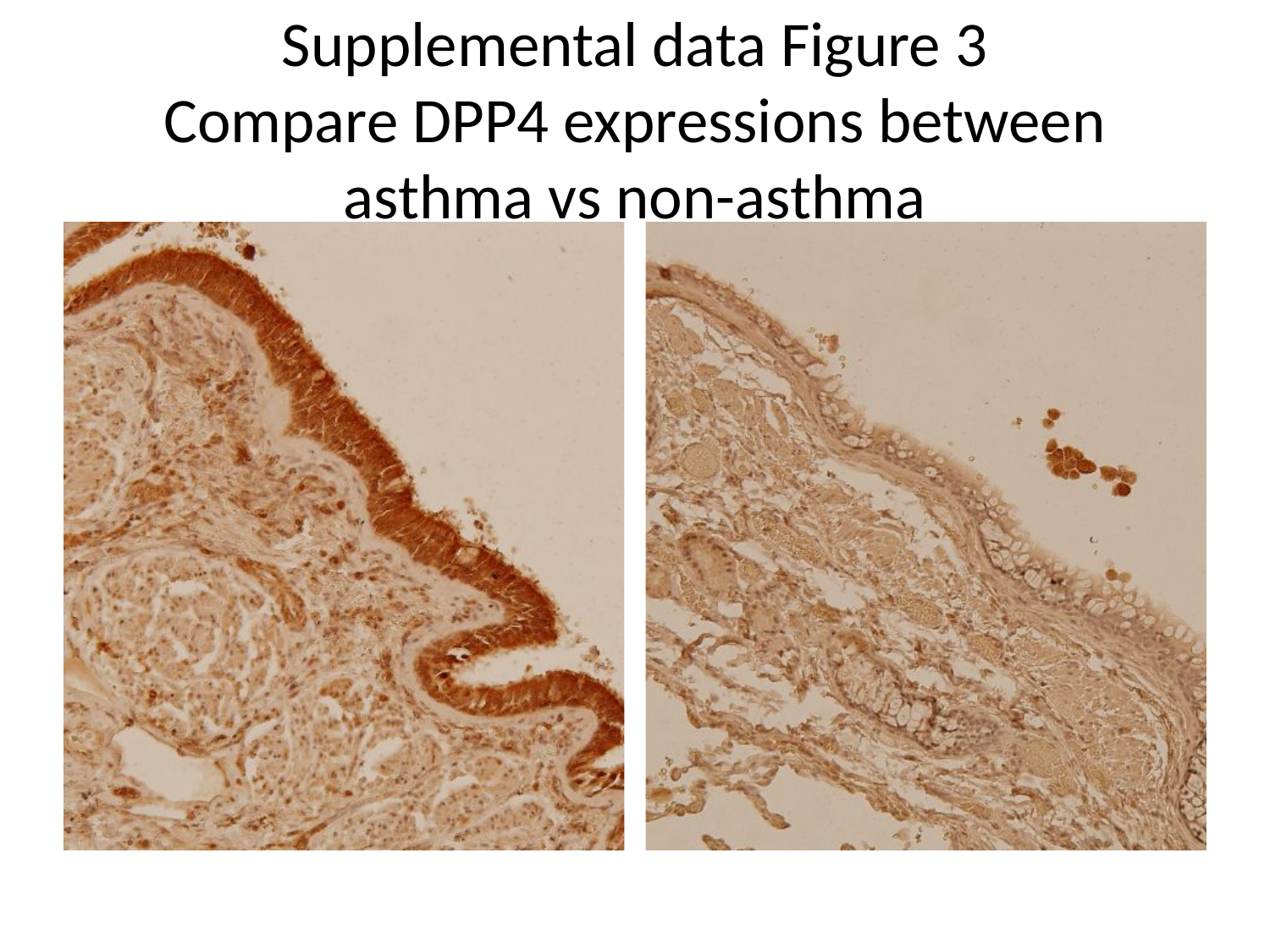

# Supplemental data Figure 3 Compare DPP4 expressions between asthma vs non-asthma

Supplement: Additional file 3: — Representative DPP4 immunostaining with surgical resected lung. The asthma group showed more staining than the non-asthma group. (PPTX 101 kb) [file 12931_2016_342_MOESM3_ESM.pptx]
