# Supplementary material for: Dipeptidyl peptidase-4 is highly expressed in bronchial epithelial cells of untreated asthma and it increases cell proliferation along with fibronectin production in airway constitutive cells
Source: Respir Res. 2016 Mar 14;17:28. doi: 10.1186/s12931-016-0342-7 (PMC4791890; doi:10.1186/s12931-016-0342-7)
Supplement: Additional file 4: — DPP4 mRNA and protein induced by IL-13 was significantly inhibited by mometasone furoate (MF). (PPTX 129 kb) [file 12931_2016_342_MOESM4_ESM.pptx]

## Slide 1
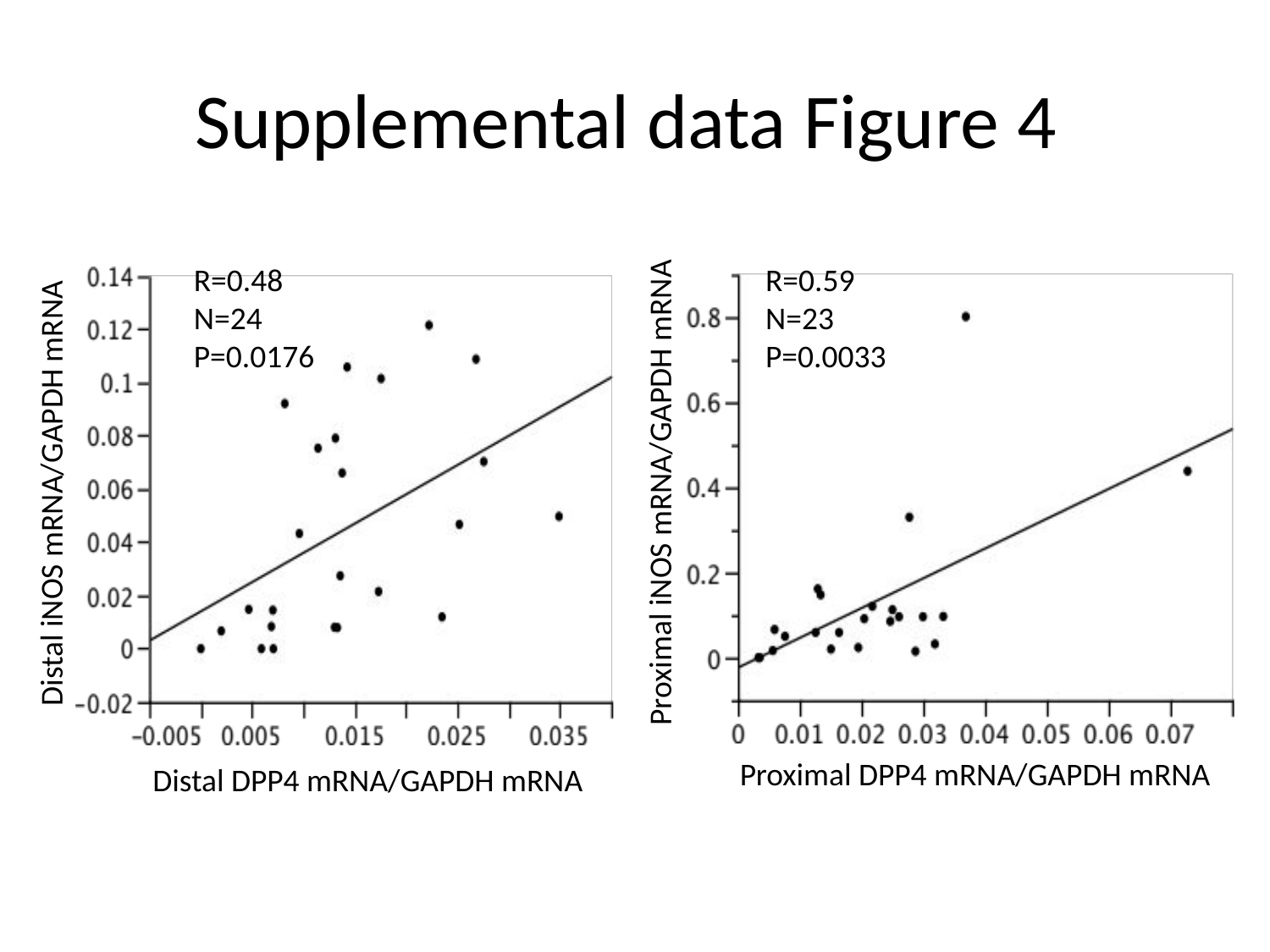

# Supplemental data Figure 4
R=0.48
N=24
P=0.0176
R=0.59
N=23
P=0.0033
Distal iNOS mRNA/GAPDH mRNA
Proximal iNOS mRNA/GAPDH mRNA
Proximal DPP4 mRNA/GAPDH mRNA
Distal DPP4 mRNA/GAPDH mRNA
